# Morphological Characteristics of Elite International Soccer Referees: Somatotype and Bioelectrical Impedance Vector Analysis

**DOI:** 10.3390/jfmk8030100

**Published:** 2023-07-24

**Authors:** Pascal Izzicupo, Cristian Petri, Sofia Serafini, Giorgio Galanti, Gabriele Mascherini

**Affiliations:** 1 Department of Medicine and Aging Science, “G. D’Annunzio” University of Chieti-Pescara, 66100 Chieti, Italy; pascal.izzicupo@unich.it (P.I.); sofiaserafini97@gmail.com (S.S.); 2 Department of Sports and Computer Science, Section of Physical Education and Sports, Universidad Pablo de Olavide, 41013 Seville, Spain; 3 Department of Experimental and Clinical Medicine, University of Florence, 50134 Florence, Italy; giorgio.galanti@unifi.it (G.G.); gabriele.mascherini@unifi.it (G.M.)

**Keywords:** soccer referees, body composition, somatotype, tolerance ellipses, DXA, BIA vector

## Abstract

This study aimed to assess the physical characteristics of elite international soccer referees, compare them with other referee populations in the literature, and establish reference tolerance ellipses for the bioelectrical impedance vector analysis (BIVA) point graph. Forty-one elite international soccer referees (age 38.8 ± 3.6 years) participated in the study. The participants underwent body composition assessments, including dual-energy X-ray absorptiometry, BIVA, and somatotype. The Somatotype Attitudinal Distance (SAD), the two-sample Hotelling’s T^2^ test and the Mahalanobis test were used to determine somatotype and bioelectrical vector differences with the literature. The average somatotype of the referees was a balanced mesomorph (2.8, 6.5, 2.8). Elite international referees significantly differed from other samples in the literature (SAD = 2.1, 2.6, 2.9 with respect to Zimbabwean, Brazilian, and South African referees, respectively). The bioelectrical vector was significantly different from the general population (T^2^ ≤ 76.6; F = 38.8; D = 1.44; *p* < 0.001) and athletes (T^2^ ≤ 25.3; F = 12.6; D = 0.8; *p* < 0.001). Somatotype values and tolerance ellipses from this study may be useful as a reference for developing training programs and improving the selection process of referees in soccer.

## 1. Introduction

In recent years, many studies have investigated the physiological and cognitive profile of soccer referees. Top-level referees generally cover a distance of 10,000–13,000 m, 10% at high intensity, and experience several accelerations at maximal or near-maximal intensity [1,2]. In addition, referees must make decisions, have control of the match, and evaluate situations under intense psychological pressure [3,4,5]. Indeed, referees exhibit diminished cardiac autonomic control from 5 h before the match up to 10 h post-match, probably due to significant psycho-physiological stress [6]. Both the physical and mental load of football refereeing require good physical fitness, of which body composition is a crucial factor [7]. However, this aspect is still underdeveloped in the literature. For example, elite referees (FIFA) show lower body mass index (BMI) and fat mass percentage than previously reported in Premier League referees [8,9], probably due to the increasing intensity of the football matches experienced in recent years. This trend was also evident in an eleven-year retrospective study on 470 Spanish elite football referees, which reported a decrease in BMI skinfolds thickness [10]. Our recent study of 43 elite international referees found a balanced mesomorph dominant somatotype pattern [11], similar to previous findings in Zimbabwean referees [12] and in contrast to results from Brazilian [13], Chilean [14], and South African [15] referees.

Interest in bioelectrical impedance vector analysis (BIVA) has recently grown in the scientific literature [16], and references have been developed for both the general [17,18] and diverse populations of athletes [19,20,21,22,23,24]. However, there is a lack of BIVA studies on soccer referees. Considering the rapid evolution of football in recent years, and how match officials’ physical and physiological profiles have changed, it is necessary to have up-to-date benchmarks, possibly on the best-performing population. Thus, the present study aimed to determine the morphological characteristics of a sample of international elite soccer referees, according to the somatotype and the BIVA methods, compare it with other samples in the literature and develop reference tolerance ellipses for the BIVA point graph. We hypothesized that the current sample would show better morphological characteristics than those already described in other studies and, for this reason, should be considered as a reference for investigators and practitioners.

## 2. Materials and Methods

### 2.1. Study Population

Using a cross-sectional design, a sample of 43 male elite international soccer referees (age 38.8 ± 3.6 years) from 6 confederations was enrolled during a seminar held in the Federal Technical Center of Coverciano (Italy) in April 2018, during the competitive season. They were designated as elite due to their registration at the highest level in the athletic federation or their receipt of financial support for their commitment to training and competitive events. Fifteen participants were from Europe, seven from South America, four from West Asia, four from Central America and two from North America, Central Asia, Oceania, West Africa and North Africa, and one from East Africa, South Africa and Est Asia, respectively.

### 2.2. Procedure

Participants underwent body composition, including dual-energy X-ray absorptiometry (DXA), somatotype and BIVA. Briefly, all the assessment were performed in the morning in a fasting condition and light clothing. Participants were required to abstain from caffeine, alcohol and exercise the day before. A certified specialist performed all the anthropometric measurements (i.e., a level 2 certification of the International Society of the Advancement of Kinanthropometry, ISAK) using a high-precision mechanical scale (SECA, Basel, Switzerland), a narrow, metallic and inextensible measuring tape (Lufkin^®^ model W606PM, London, UK; precision = 1 mm), a skinfold caliper (Holtain Ltd., Crymych, UK; precision = 0.2 mm) and a sliding caliper (GMP, Zürich, Switzerland), for body mass and standing height, body girths, skinfolds and bone diameters, respectively. The sum of six skinfolds was calculated as the sum of triceps, subscapular, supraspinale, abdominal, thigh and calf skinfolds, while selected anthropometric measures were used to determine somatotype following the methods of Carter and Heath [25].

Bioimpedance analysis was performed by a phase sensitive device (BIA 101 Anniversary AKERN srl, Florence, Italy) working with alternating sinusoidal electric current of 400 microampere at an operating frequency of 50 kHz (±1%). The device was calibrated before assessment using the standard control circuit supplied by the manufacturer with a known impedance (resistance (R) = 380 ohm; reactance (Xc) = 45 ohm). The accuracy of the device was 0.1% for R and 0.1% for Xc. For the bioelectrical impedance measurement, each participant was lying in the supine position for at least two minutes, with a leg opening of 45° compared to the median line of the body and the upper limbs, distant 30° from the trunk. Very low-intrinsic-impedance (<30 ohm) disposable electrodes (Biatrodes™ Akern Srl; Florence, Italy) were placed on the right side at metacarpal and metatarsal sites of the right wrist and ankle. Measurements were made on an isolated cot from electrical conductors [26]. Bioimpedance vector analysis was carried out using the BIVA method, normalizing resistance (R) and reactance (Xc) parameters for H in meters [26]. Bioelectrical phase angle (PhA) was calculated as the arctangent of Xc/R × 180°/π [27].

A DXA scanner (Hologic QDR Series, Delphi A model, Bedford, MA, USA) with Hologic APEX software (Version 13.3:3, Hologic, Bedford, MA, USA), was used to estimate FM%. The instrument was calibrated with phantoms as per the manufacturer’s guidelines each day prior to measurements. Participants assumed a stationary supine position on the scanning table. All scanning and analyses were performed by the same technician to ensure consistency and in accordance with standardized testing protocols recognized as best practice [28,29,30]. The study was designed and conducted in accordance with the Helsinki Declaration. The ethics committee of the Italian Football Association approved this study and all the participants signed written informed consent prior to their inclusion in the study (approval code: 03032018).

### 2.3. Statistical Analysis

The normality of the data was verified by applying the Shapiro–Wilk test, and descriptive statistics were calculated for each independent variable. All variables followed the Gaussian distribution. Two European participants were excluded from the final sample due to the lack of bioelectrical parameters (n = 41). Each participant was plotted into the somatochart and the relative somatotype was calculated. The somatotype attitudinal distance (SAD) was calculated to compare the current sample with first-division Zimbabwean referees and assistant referees [12] and national- and regional-level Brazilian referees [13]. A SAD ≥ 2 was assumed as a distance statistically different between two somatotype means [25]. Each participant was plotted in the tolerance ellipses (50%, 75% and 95%) of the Italian reference population [26]. A two-sample Hotelling’s T^2^ test was used to determine the impedance vector differences with respect to the reference population [26] and the athletic population [17], as well as to determine differences between somatotypes. Distances between ellipses were calculated by the Mahalanobis test [31]. A *p*-value < 0.05 was considered significant. BIVA software [31] was used to plot and compare the bioelectrical parameters and compute the tolerance ellipses (50%, 75% and 95%) of the investigated sample.

## 3. Results

**Figure 1:**
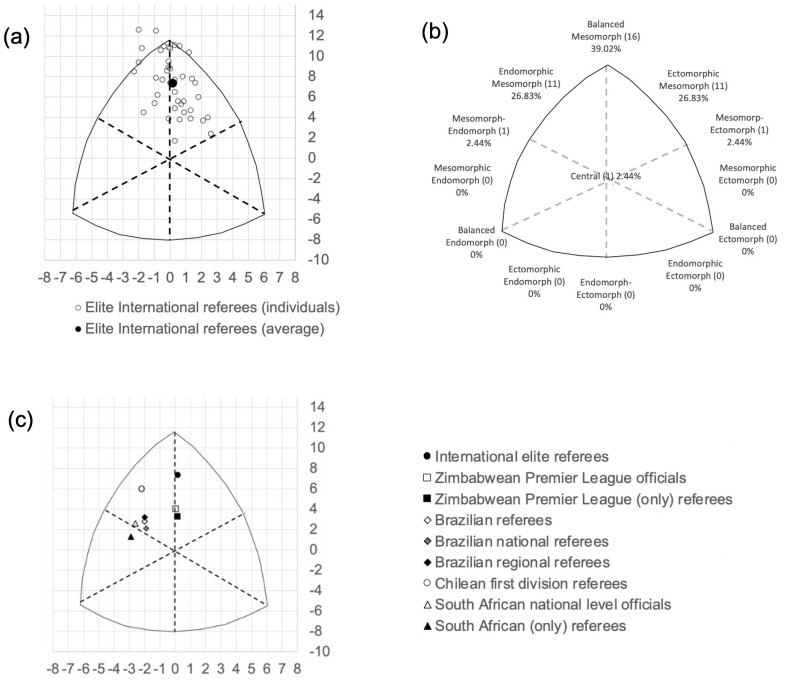
(**a**) Somatotype distribution of the international elite male soccer referees; (**b**) somatotype categories of the international elite male soccer referees; (**c**) comparison with other published references [12,13,14,15].

Table 1 describes the general, anthropometric and bioelectrical characteristics of the sample. The average and most represented somatotype (n = 16) was balanced mesomorph, followed by endomorphic mesomorph (n = 11) and ectomorphic mesomorph (n = 11) (Figure 1a,b). Mesomorph–endomorph, mesomorph–ectomorph and central somatotypes comprised only one individual for each category.

The current sample showed a better somatotype than other referee populations in the literature (Figure 1c). Compared to first-division Zimbabwean referees and assistant referees, both samples were, on average, balanced mesomorph, but the values of mesomorphy were higher in the present study. The SAD value of 1.9 was slightly lower than the values considered statistically significant (≥2), according to Carter and Heath [25]. However, the SAD becomes significant, increasing from 1.9 to 2.1, if only the referees are considered in Banda at al.’s [12] sample (i.e., excluding assistant referees). Compared to the Brazilian national- and regional-level referees, the current sample was significantly different (SAD = 2.6), with lower endomorphy and both higher ectomorphy and mesomorphy. Similar results were observed by dividing the Brazilian sample into national- (SAD = 2.9) and regional (SAD = 2.5)-level referees [13]. While the difference was not statistically significant (SAD = 1.8), Chilean referees in the sample had an average somatotype of endomorphic mesomorphs, which contrasts with the dominant balanced mesomorph somatotype of elite international referees [14]. On the other hand, South African officials were very far from the current sample, with a dominant mesomorph–endomorph somatotype and a SAD value of 2.9 [15]. However, the South African referees subsample was even more distant from the international counterpart in the present sample, with a dominant mesomorphic endomorph somatotype (SAD = 3.4).

**Figure 2:**
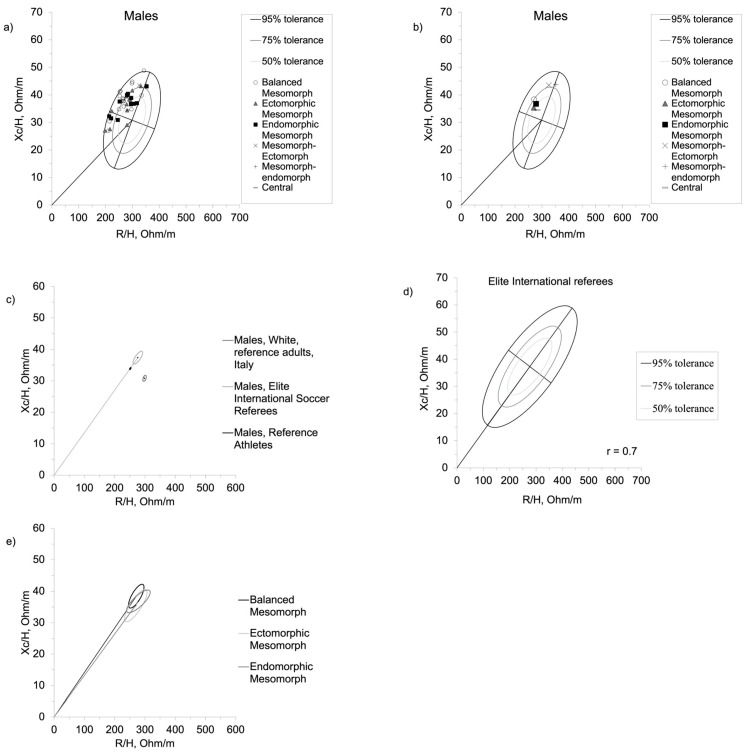
(**a**) Scattergram of the international elite soccer referees, according to the somatotype category, within the BIVA point graph with the 50%, 75% and 95% tolerance ellipses of the general population; (**b**) the average position of the different somatotypes; (**c**) comparison with the general [24] and athletic references [17]; (**d**) 50%, 75% and 95% tolerance ellipses derived from the current sample; (**e**) comparison among the different identified somatotypes that characterized elite referees.

The BIVA point graph indicates that elite international referees mainly fell in the upper left quadrant (Figure 2a). Figure 2b shows the average position on the BIVA point graph for each somatotype of the current referee population. Compared with the general male population [26] and athletic reference [17], elite international referees are statistically different. In particular, they exhibited a greater tendency to fall towards the left and upward compared to the general population (T^2^ ≤ 76.6; F = 38.8; D = 1.44; *p* < 0.001), and towards the right and upward compared to the athletic population (T^2^ ≤ 25.3; F = 12.6; D = 0.8; *p* < 0.001) (Figure 2c). The tolerance ellipses representing their specific 50%, 75% and 95% ranges are shown in Figure 2d. Furthermore, Figure 2e compares referees with a balanced mesomorph, endomorphic mesomorph and ectomorphic mesomorph physique, but no significant statistical differences were found.

## 4. Discussion

The present study describes a sample of international elite soccer referees. The first important result is that international soccer referees showed a different somatotype than those previously presented in the literature, with the highest mesomorphic component and lower endomorphy [8,9,10]. They can be considered as a reference for practitioners. Secondly, they showed bioelectrical impedance characteristics that are significantly different from the general and the athletic populations [17,26]; specific tolerance ellipses for soccer referees were developed.

In recent years, the intensity of football matches has increased, leading to a corresponding increase in soccer refereeing intensity. Consequently, the relative fat mass of soccer referees was lower in more recent samples compared to those previously assessed [8,9,10]. In the present study, the value of fat mass percentage is higher than that of other studies. Zimbabwean (12.0 ± 2.6%), Greek (16.7 ± 4.5%) and South African (12.6 ± 4.2%) referees showed lower relative fat mass values, while only Brazilian referees (18.5 ± 4.3%) had higher values compared to the present sample [12,13,15,32]. However, this difference can be explained by considering that the studies mentioned above performed the body composition assessment using the anthropometric method. At the same time, the present fat mass percentage value is derived from DXA [11]. As previously reported, the fat mass values of the current population are underestimated by the commonly used fat mass equations (range 11.0–15.2%). Thus, caution should be applied when considering fat mass percentage values in the literature derived from equations developed for the general population, regardless of the method adopted (e.g., skinfolds, bioelectrical impedance analysis). For example, Matias et al. [33] developed a bioimpedance-based model for fat-free mass prediction based on the four-compartment model in a sample of national level athletes that can be used to overcome problems arising when measuring adult elite athletes. Similar considerations can be raised for other compartments such as muscle mass [34] and total body water and its distribution into the intra- and extra-cellular compartment [35]. From this point of view, a solution is to adopt specific equations for the investigated population [11]. Alternatively, this problem can be avoided using raw skinfold values, like the sum of six or seven skinfolds. Despite the higher value of FM%, the current sample showed the lowest sum of the six skinfolds when compared with other samples in the literature [12,36], confirming that FM% was underestimated in previous studies on soccer referees. Reilly and Gregson [8] indicated a value of FM of 13% in football players using the DXA technique, and other authors also found values lower than 11% [37,38]. This difference with our referees’ sample may be ascribed to the lower level of performance required from referees compared to players. However, we should also consider that elite referees are generally older than other athletes. Indeed, at the elite level, the age range is between 35 and 40 years [3,39,40]. Fernandez Perez et al. [13] indicated as Brazilian regional-level referees had lower fat values than the national ones, probably due to the age difference. Furthermore, a longitudinal study on Brazilian football referees found that their mean body fat percentage increased from 13.2 ± 2.9% to 17.3 ± 3.9 in ten years, moving from a balanced mesomorph pattern to a mesomorph–endomorph pattern [41]. The authors suggest that elite referees are not professionals like elite soccer players, and their training sessions and nutrition are less planned than players.

Previous studies have investigated the somatotype of soccer referees. Although the mesomorph component was dominant across the samples in the literature, Brazilian [13] and Chilean [14] referees also showed an important contribution of the endomorph component. On the other hand, Zimbabwean [12] and South African [15] referees showed a balanced mesomorph dominant somatotype, like in the current study. These differences can be explained by considering the above considerations about fat mass, like the role of refereeing intensity and age. Furthermore, geographical ancestry can also play a role. For example, studies in the international context have identified group differences in somatotype in Australia [42], New Zealand [43] and the United Kingdom [44] as well as in adult body mass index–adiposity relationships [45]. From this point of view, South American referees [13,14] seem to have more fat than African ones [12,15]. In the present study it was not possible to compare referees based on the geographical ancestry, due to the small size of each sub-group that in some cases were represented by only one participant. Notwithstanding, a clear tendency can be observed for a somatotypes with a prevalent mesomorph component for all geographical groups (see Figure S1 in the Supplementary Material).

Compared to the previous literature, our sample is up to date. International elite referees showed a significant distance on the somatochart, characterized by a higher mesomorphy, compared to their Zimbabwean, Brazilian, Chilean and South African counterparts, which indicates them as the fittest. This difference can be attributed to both the higher competitive level of the current sample and to the evolution of playing intensity experienced by elite referees. Soccer players show a dominant balanced mesomorph morphology, and our results indicate that soccer referees are becoming closer to their players’ counterparts [46]. We also found other somatotypes, particularly endomorphic mesomorph and ectomorphic mesomorph, but such variability can be found even in soccer players. Additionally, the current sample showed a dominant mesomorph component which is a determinant for high-level performance, regardless of sports discipline.

Regarding the BIVA, elite international referees mainly fell in the upper left quadrant (Figure 2a). Furthermore, some referees fell in the lower left quadrant area to the left of the impedance vector, as previously indicated by Campa for the athletic population [47]. The current sample was characterized by bioelectrical impedance parameters indicating higher cellular mass than the general population, but lower than other athletes (Figure 2c). This result suggests that soccer referees are a specific population with peculiar bioelectrical properties that differentiate them from the general population, putting them more to the left and up, but also from the athletic population, which shows a shorter vector positioned even more to the left. Thus, although soccer referees are characterized by bioelectrical values associated with better cellular health and body composition than the general population, they are not at the same level as other athletes. Finally, we did not find differences in the bioelectrical vector between different somatotypes (Figure 2e). This result suggests that, despite different morphologies, referees are comparable regarding those parameters that indicate cellular health, hydration and nutritional status. As well as for somatotype, similar consideration can be done for the BIVA regarding the geographical ancestry of the referees in the current sample. Previous studies indicate differences in the impedance vector distribution for populations from different geographical ancestry [48,49,50]. However, the small size of each sub-group does not allow a statistical comparison (see Figure S2 in the Supplementary Material). The current sample was assessed in April, during the competitive season. In a sample of male elite soccer players, Mascherini et al. [51] showed impedance vector length changes during the competitive season, indicating fluid gains during the pre-season, probably due to the plasma volume expansion and increase in glycogen stores, and fluid loss in the mid-season as indicated by the increase in R/h, accompanied by the increase in PhA that indicates gains in body cell mass. Thus, the different components of BIVA are not equally informative during the season, also considering that different workouts can determine different acute adaptations for the same training volume [52] and that the training approaches can vary greatly in intensity and volume for similar results [53]. Furthermore, the role of off-training behaviors in body composition should not be overlocked. It can be speculated that the current sample was characterized by high body cell mass and low body fluids at the moment of the assessment compared to the previous months. Thus, when comparing groups assessed in different season phases, caution must be taken.

The present study has some strengths. Firstly, the sample comprised the best referees at the international level and was assessed on a single occasion. Secondly, fat mass was measured using the gold standard method (i.e., DXA). Thirdly, the study provides a comprehensive description of an elite sample using multiple methods (i.e., DXA, Anthropometry and BIVA), which can serve as a useful reference for researchers and practitioners. It is important to underline that body composition assessment can vary considerably in the methods but also according to instruments and the techniques adopted. Bioelectrical impendence analysis can be performed using single and multi-frequency modes, as well as hand-to-hand, foot-to-foot, foot-to-hand, and segmental techniques [24,54]. This makes it difficult to compare measurements taken with different instruments and, therefore, they are not generalizable for Bioelectrical impendence analysis technologies. Finally, the sample is the most up to date among the literature on elite soccer referees, providing insights into the evolution of soccer match intensity. However, the study is limited by its descriptive nature, which only allows for hypothesis generation regarding considerations such as soccer evolution. Additionally, the study did not collect information on the referees’ training routines.

## 5. Conclusions

In summary, our findings suggest that elite soccer referees, based on their body composition, somatotype and bioelectrical characteristics, are more comparable to soccer players than the general population. As referees are expected to meet high fitness standards, they should be considered athletes, even if they are not professional like their player counterparts. During a game, referees cover an average distance of 10,000–13,000 m [1,2] and expend an estimated 4700 to 5600 kJ (1120–1340 kcal) [55,56]. Similarly, soccer players cover a similar total distance during matches, but with variations based on tactical position and match demands [57]. Our study implies that elite soccer referees are in good physical condition yet have the potential for further improvement through targeted training and nutrition plans. The current sample comprised the best international referees and is the most up-to-date in the literature, at least in consideration of the high level. For this reason, both bioelectrical tolerance ellipses and somatotype profiles from the current study can be used by coaches, trainers and researchers as a reference. Furthermore, the equation of Petri et al. derived from the present sample [11] can be used for fat mass estimation in soccer referees. Overall, the evidence from this study suggests that soccer referees have margins of improvement in body composition and provides the instruments needed for evaluating and monitoring it.

## Figures and Tables

**Table 1:** General, anthropometric and bioelectrical characteristics of the international-level elite referee.

Variable	Mean	SD	Minimum	Maximum
Age (years)	38.8	3.6	29.5	44.1
Body mass (kg)	75.6	6.7	61.0	94.0
Standing height (cm)	180.6	6.1	171.0	194.0
Body mass index (kg/m^2^)	23.2	1.4	20.6	25.8
Sum of six skinfolds (mm)	60.3	16.4	34.0	112.6
Endomorphy	2.8	0.9	1.2	5.1
Mesomorphy	6.5	1.2	4.2	8.5
Ectomorphy	2.8	0.7	1.2	4.6
Fat mass (%)	18.4	4.1	11.5	28.0
Fat mass (kg)	13.5	3.1	8.7	21.6
Resistance (Ω)	498.6	69.0	380.0	622.1
Reactance (Ω)	67.4	8.6	49.9	83.9
Resistance/height (Ω/m)	276.3	39.5	197.9	352.9
Reactance/height (Ω/m)	37.4	5.1	27.0	48.8
Phase angle (°)	7.7	0.7	5.9	9.2

The table describes the average values, standard deviation (SD), minimum and maximum values for the general, anthropometric and bioelectrical characteristics of the international-level elite referee. Sum of six skinfolds = the sum of triceps, subscapular, supraspinale, abdominal, thigh and calf skinfolds.

## Data Availability

The raw data supporting the conclusion of this article will be made available by the authors, based on a reasoned request.

## Supplementary Materials

The following supporting information can be downloaded at: https://www.mdpi.com/article/10.3390/jfmk8030100/s1, Figure S1: Somatotype of male elite international soccer referees, according to geographical origin; Figure S2. Bioelectrical impedance vector of male elite international soccer referees, according to geographical origin.
