# Supplementary material for: Morphological Characteristics of Elite International Soccer Referees: Somatotype and Bioelectrical Impedance Vector Analysis
Source: J Funct Morphol Kinesiol. 2023 Jul 24;8(3):100. doi: 10.3390/jfmk8030100 (PMC10366770; doi:10.3390/jfmk8030100)
Supplement: Supplementary file 1 [file jfmk-08-00100-s001.zip › jfmk-2500335-supplementary.pdf]

Supplementary Material

Figure S1. Somatotype of male elite international soccer referees, according to geographical origin.

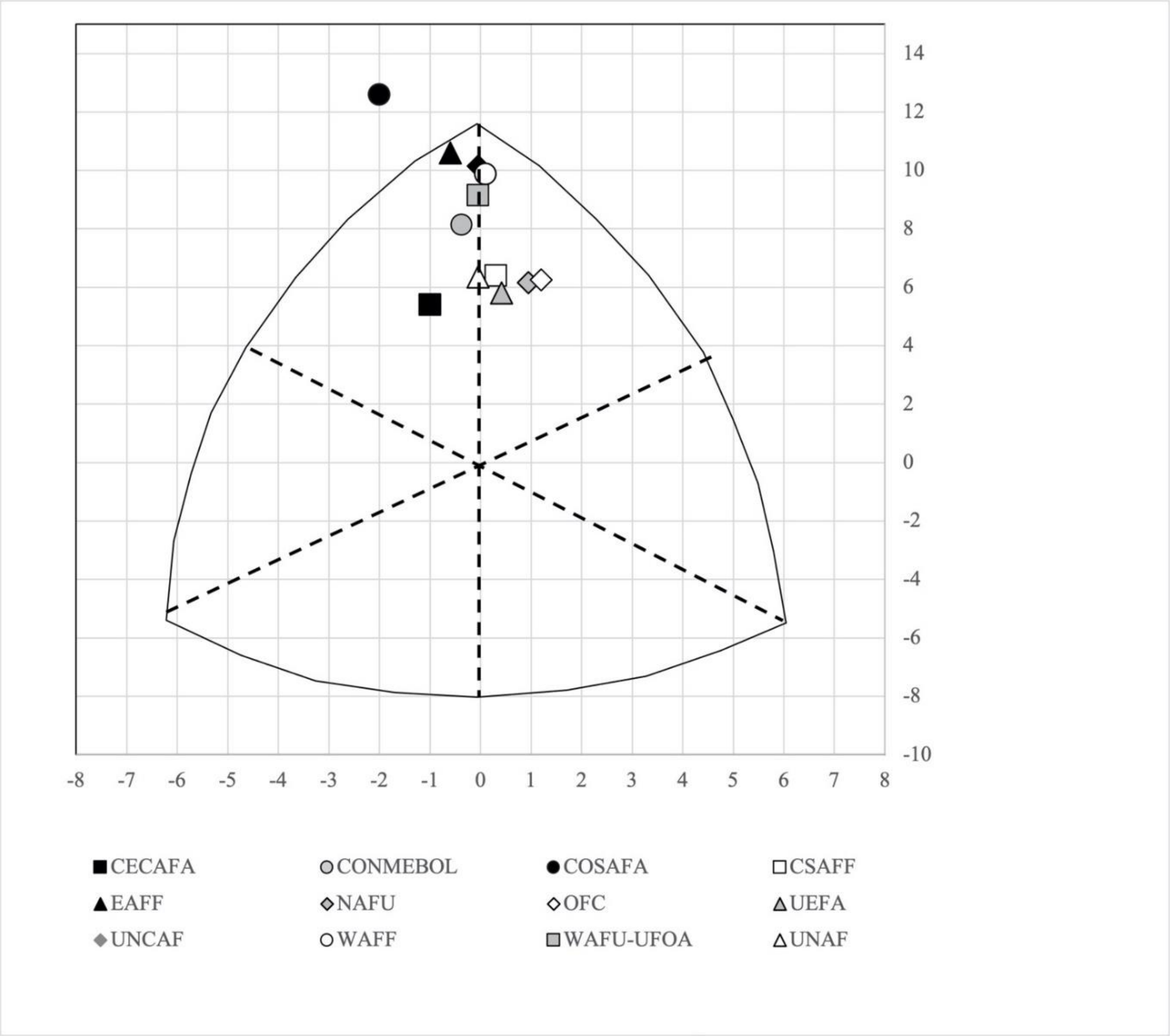

Figure S1. CECAFA = Central and East Africa Football Associations; CSAFF = [16:12] Council for East and Central Africa Football Associations; UEFA = Union of European Football association; UNAF = Union of North African Football Federations; EAFF = East Asian Football Federation; UNCAF = Unión Centroamericana de Fútbol; CONMEBOL = Confederación sudamericana de Fútbol; NAFU = North American Football Union; WAFF = West Asian Football Federation; COSAFA = Council of Southern Africa Football Associations; OFC = Oceania Football Confederation; WAFU-UFOA = West Africa Football Union;

Figure S2. Bioelectrical impedance vector of male elite international soccer referees, according to geographical origin.

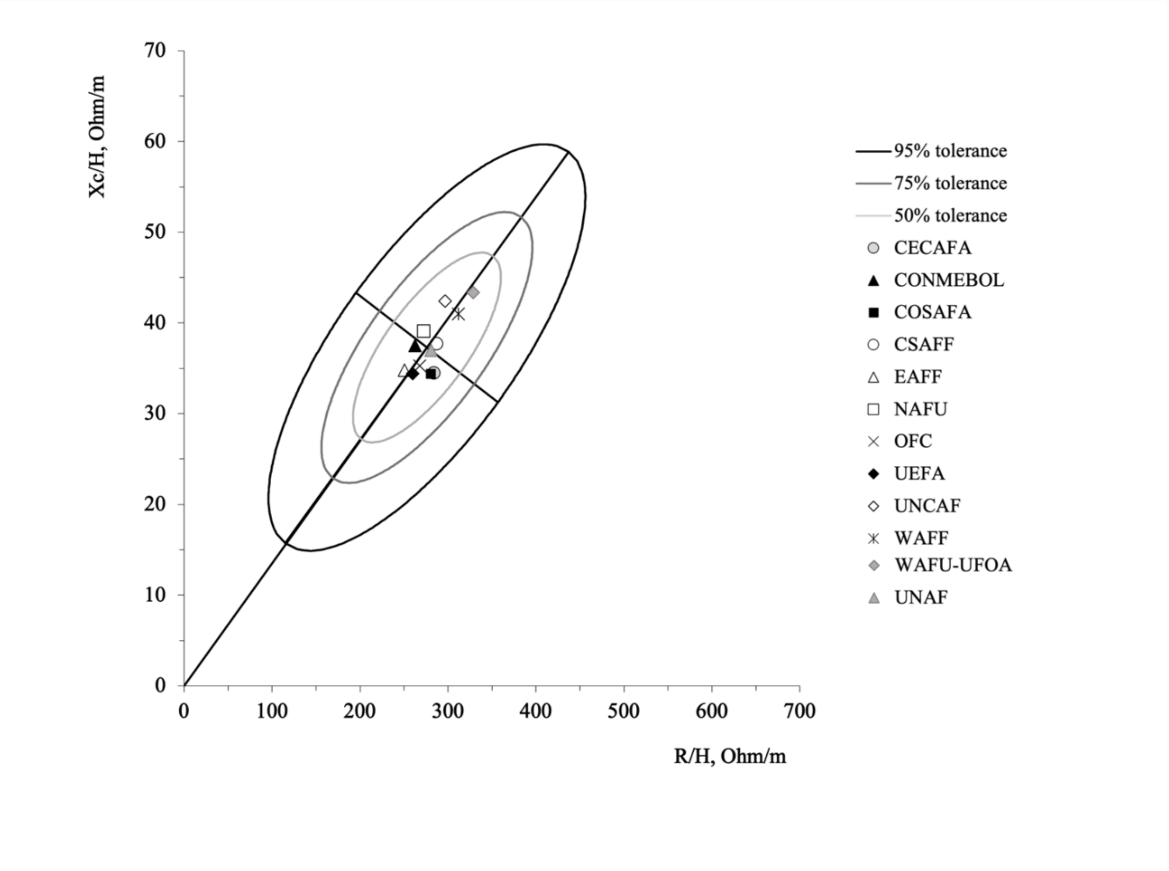

Figure S2. CECAFA = Central and East Africa Football Associations; CSAFF = [16:12] Council for East and Central Africa Football Associations; UEFA = Union of European Football association; UNAF = Union of North African Football Federations; EAFF = East Asian Football Federation; UNCAF = Unión Centroamericana de Fútbol; CONMEBOL = Confederación sudamericana de Fútbol; NAFU = North American Football Union; WAFF = West Asian Football Federation; COSAFA = Council of Southern Africa Football Associations; OFC = Oceania Football Confederation; WAFU-UFOA = West Africa Football Union;
